# On the detection of population heterogeneity in causation between two variables: Finite mixture modeling of data collected from twin pairs

**DOI:** 10.21203/rs.3.rs-4576809/v1

**Published:** 2024-07-13

**Authors:** Philip Vinh, Brad Verhulst, Conor V Dolan, Michael C Neale, Hermine HM Maes

**Affiliations:** Virginia Commonwealth University; Texas A&M University; Vrije Universiteit Amsterdam; Virginia Commonwealth University; Virginia Commonwealth University

**Keywords:** Causality, Mixture modeling, Twin design, Statistical Modeling

## Abstract

Causal inference is inherently complex, often dependent on key assumptions that are sometimes overlooked. One such assumption is the potential for unidirectional or bidirectional causality, while another is population homogeneity, which suggests that the causal direction between two variables remains consistent across the study sample. Discerning these processes requires meticulous data collection through an appropriate research design and the use of suitable software to define and fit alternative models.

In psychiatry, the co-occurrence of different disorders is common and can stem from various origins. A patient diagnosed with two disorders might have one recognized as primary and the other as secondary, suggesting the existence of two types of comorbidity within the population. For example, in some individuals, depression might lead to substance use, while in others, substance use could lead to depression.

Identifying the primary disorder is crucial for developing effective treatment plans. This article explores the use of finite mixture models to depict within-sample heterogeneity. We begin with the Direction of Causation (DoC) model for twin data and extend it to a mixture distribution model. This extension allows for the calculation of the likelihood of each individual’s data for the two alternate causal directions. Given twin data, there are four possible pairwise combinations of causal direction. Through simulations, we investigate the Direction of Causation Twin Mixture (mixCLPM) model’s potential to detect and model heterogeneity due to varying causal directions.

## Introduction

Determining whether a potential risk factor causes a disease is crucial for developing effective treatments or interventions. While randomized experiments, including randomized controlled trials, are widely used to establish causal relationships, they are often infeasible or unethical in many scenarios and cannot rule out reverse causal effects. In such situations, it is necessary to use quasi-experimental or correlational designs to illuminate causal processes (Shadish et al., 2002). However, drawing valid causal conclusions in non-experimental approaches is challenging because causal inference relies on strong assumptions that can be difficult to test. Additionally, causal effects may vary between individuals within the population.

For example, the high co-occurrence of depression and alcohol use disorder (AUD) (Grant & Harford, 1995; Hasin et al., 2018) may arise from multiple distinct reasons. Some individuals with depression may use psychoactive substances to alleviate their symptoms (i.e., self-medication; Polimanti et al., 2019), while others who frequently drink excessively may develop symptoms of depression (Fergusson et al., 2009). Another possibility is that both disorders result from a shared pathophysiology (Zhou et al., 2017). These different explanations for the association between substance use and depression may coexist within a population, resulting in heterogeneous causal pathways. Our goal is to examine the feasibility of a research design and statistical method that can disentangle these complex heterogeneous causal relationships. To this end, we have adapted the Direction of Causation (DoC) model (Gillespie et al., 2003; Heath et al., 1993; Maes et al., 2021; McAdams et al., 2021; Neale & Cardon, 1992; Verhulst & Estabrook, 2012) with finite mixture modeling to allow for heterogeneity in the population.

### The Twin Direction of Causation (DoC) Model

One of the most popular research designs for tackling nature versus nurture questions is the classical twin study (CTS). In this approach, pairs of monozygotic (MZ) twins and dizygotic (DZ) twins are measured on the same traits. Greater covariance of MZ pairs is expected if genetic factors influence the trait, since members of an MZ twin pair have almost identical genomes, whereas DZ pairs’ genomes correlate on average by one half.

In behavior genetics, multivariate modeling can be used to study the covariance or comorbidity among phenotypes. Previous research has demonstrated that cross-sectional data collected from a CTS can be used to test hypotheses concerning the causal relationship between two traits. The univariate twin model decomposes phenotypic variance into genetic and environmental sources, and the multivariate model similarly decomposes phenotypic covariance. This decomposition provides insight into the genetic and environmental contributions to phenotypic covariance, or comorbidity in the case of disorders.

The bivariate twin data can be used to investigate the direction of causality. Given certain assumptions, the Direction of Causation (DoC) twin model can reject specific hypotheses concerning unidirectional or bidirectional causal relationships. The key information needed for this are the cross-twin cross-trait correlations (Duffy & Martin, 1994; Heath et al., 1993; Maes et al., 2021; McAdams et al., 2021; Neale & Cardon, 1992). For instance, if “X causes Y,” the pattern of MZ and DZ cross-twin cross-trait covariances is predicted to be similar to the twin correlations for X (the causal trait). The DoC model may include five potential sources of covariance: 1) the additive genetic covariance (*r*_*A*_); 2) the shared environmental covariance (*r*_*C*_); 3) the unshared environmental covariance (*r*_*E*_); 4) the causal effect of the first trait on the second; or 5) the causal effect of the second trait on the first. However, due to model identification limitations, only three of these five parameters can be estimated in any single analysis.

For any two traits with sufficiently different inheritance patterns, the DoC model can be used to investigate the direction of causation. Suppose, for example, trait X consists of additive genetic (A) and unshared environment (E) components, while trait Y consists of shared (C) and unshared (E) environmental components. If X causes Y, the cross-twin, cross-trait covariance will reflect the heritable component in X and the within-person covariance between X and Y. By contrast, if Y causes X, the cross-twin, cross-trait covariance would mirror the common environment component in Y. The larger the difference in the modes of transmission between the two traits, the easier it will be to distinguish the causal direction. In practice, both traits may be influenced by all three sources of variance, and as long as the proportions of variance differ and there is a sufficient sample size, it is possible to reject some of the simpler models (Heath et al., 1993). Therefore, the DoC model provides an effective method for illuminating causal effects in cross-sectional twin data.

### Finite Mixture Modeling to Understand Causality in Heterogeneous Populations

Most causal modeling, including the DoC model, involves fitting alternative models to data under the assumption of homogeneity. This assumption implies that the putative causal model holds for all individuals in the sample (and by extension, the population). The statistical counterpart of this assumption is that the observations are identically distributed (Brand & Thomas, 2013; Xie, 2013). The homogeneity of variance assumption rules out the possibility that the causal associations between comorbid traits differ between subpopulations. Multiple causal processes within the same population imply causal heterogeneity, violating the identical distribution assumption. Failure to account for causal heterogeneity may give rise to parameter bias and incorrect and inconsistent causal inferences (Muthén, 1989).

The role of causality in comorbidity is difficult to ascertain because comorbidity may be due to shared risk factors (confounding), direct causal relationships, or both. In the case of causal heterogeneity, these are not mutually exclusive (Neale & Kendler, 1995). Across psychiatric disorders, comorbidity is often the rule rather than the exception (Kessler et al., 1994; McGrath et al., 2020). If the association between comorbid disorders (e.g., X and Y) is causal, but the direction of the causal relationship varies as a function of latent individual characteristics, the bivariate distribution is a mixture distribution of two distributions. A proportion, ω_i_, of the population would comprise individuals in whom X causes Y, while the remaining proportion, 1- ω_i_, would comprise individuals in whom Y causes X. Modeling this type of heterogeneity can be done by means of finite mixture modeling (Dolan & van der Maas, 1998; Lubke & Muthén, 2005; McLachlan & Peel, 2000; Vermunt & Magidson, 2014; Yung, 1997).

## Materials and Methods

**Figure 1:**
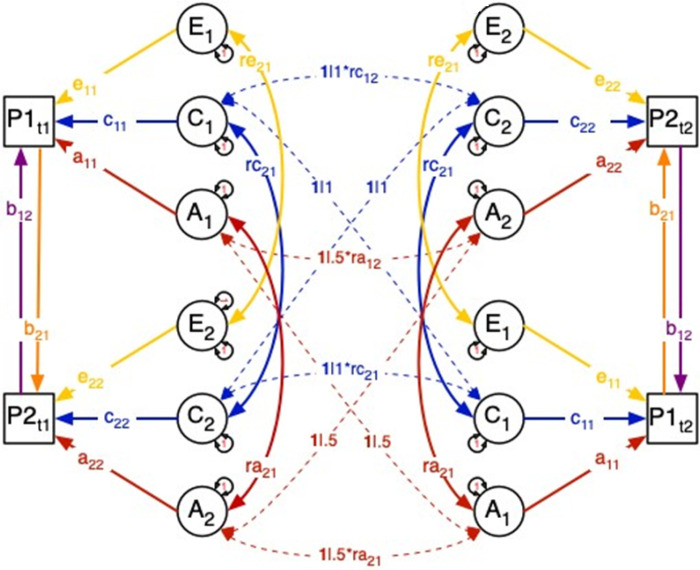
Path diagram representation of the Direction of Causation twin model. The model as shown is not identified. Diagram is drawn with standard structural equation modeling symbology where circles represent latent variables, squares represent observed variables, double-headed paths represent covariance/variance, and single headed arrows represent regressions.

Figure 1 shows a path diagram of the DoC model, including all possible paths between two traits that can be estimated using cross-sectional twin data. In a bivariate ACE model, each trait is influenced by latent additive genetic (*A*), shared environmental (*C*), and unique environmental (*E*) variance components which may covary between the traits (parameters *r*_*A*_, *r*_*C*_, and *r*_*E*_). Causal effects between X and Y are represented by the direct regression paths between the phenotypes (parameters b1 and b2). Due to identification constraints, only three of the five potential sources of covariance (*b*_*12*_, *b*_*21*_, *r*_*A*_, *r*_*C*_, and *r*_*E*_) can be estimated in any model (Heath et al., 1993; Neale & Cardon, 1992). Further, the model makes the standard assumptions of non-random mating, the absence of non-additive genetic variation, and gene-environment independence (Neale & Cardon, 1992). Differences in the patterns of the cross-twin, cross-trait covariances enable testing causal hypotheses.

The likelihood ratio test is typically used to compare the model fit of the DoC and other variance component models. Unidirectional DoC models are nested within the Cholesky ACE model, so likelihood ratio testing is appropriate when certain regularity conditions are met (Steiger et al., 1985; Verhulst et al., 2019). When models are not nested, such as the bidirectional DoC model with additive genetic correlation (*r*_*A*_ estimated, *r*_*C*_ and *r*_*E*_ fixed) versus one with unshared environment correlation (*r*_*E*_ estimated and *r*_*A*_ and *r*_*C*_ fixed), a parsimony-based fit index such as Akaike Information Criterion (AIC) can be used. The AIC, calculated as twice the negative log-likelihood minus twice the degrees of freedom, balances model complexity and model fit to achieve a parsimony-related statistic. The model with the lowest AIC is the model of choice, in principle. Although the difference in log-likelihood between models with different numbers of mixture components may not meet the Steiger et al. (1985) regularity conditions, and hence may not be distributed as chi-squared, it is still useful as a guide to relative model fit.

### Integrating Mixture Modeling with the Direction of Causation Model

Given that the CTD offers the means to address the direction of causation, it seems plausible that a mixture distribution model may be able to detect population heterogeneity in this respect. Finite mixture modeling of twin data has been considered before. However, this was to conduct genetic covariance structure analysis in the absence of zygosity information (Benyamin et al., 2006; Neale, 2003), or to investigate (latent) group differences in genetic and environmental variance components (Gillespie & Neale, 2006). The present aim is to investigate the feasibility of mixture modeling to detect causal heterogeneity. Accordingly, we conducted a simulation study to: i) ensure that the mixture model accurately recovers parameter values used to simulate the data; ii) compare the model fit statistics between mixture and non-mixture DoC models; and iii) evaluate the posterior probabilities that each individual belongs to each class (i.e., the X-causes-Y class or the Y-causes-X class). Thus, we aim to determine the feasibility of finite mixture modeling of heterogeneity of causal relations using twin data, estimate the required sample sizes, and identify patterns of parameters that aid in model fitting.

### Structural Equation Mixture Modeling (SEMM)

Here we formally define the finite mixture distribution model. Let y denote a column vector of continuous observations, and *C* denote the number of mixture classes. The prior probability of belonging to latent class $i,\;\omega_{i}$, is equal to the proportion of the population belonging to that latent class. In a mixture model, the probability density function with C latent classes can be expressed as:

(1) $$f\left(\backslash \mathrm{varvec}y\right)=\sum_{i=1}^{C}\omega_{i}\Phi_{i}\left(y;\theta_{i}\right)$$

where $\Phi_{i}$ is the *i-th* density, $\theta_{i}$ is the vector of the parameters of the *i-th* density, and, as, above, $\omega_{i}$ is the corresponding mixture proportions, where $\sum \omega_{i}=1$. In the DoC mixture model, the class-specific densities $\Phi_{i}\;(y;\;\theta_{i})$ come from the same parametric family (multivariate normal) with class-specific mean and covariance matrices, which are a function of the $\theta_{i}$ parameter estimates. Combining structural equation modeling (SEM) with finite mixture modeling, known as Structural Equation Mixture Modeling (SEMM), involves specifying an SEM structure within each class (Dolan & van der Maas, 1998; Lubke & Muthén, 2005; Vermunt & Magidson, 2014; Yung, 1997). The SEM structures must differ in some way to ensure identification of the mixture proportions. This approach allows for the estimation of: i) the number of classes, ii) the parameters of the densities and the mixture proportions, and iii) each individual’s conditional class membership (posterior) probabilities.

### Direction of Causation Twin Mixture Model (mixDoC)

**Figure 2:**
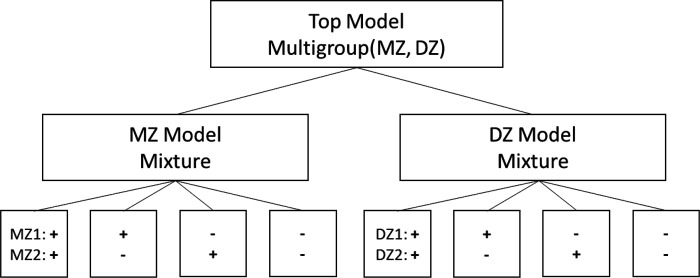
Schematic Diagram of the Mixture Direction of Causation (mixDoC) Twin Model. Figure illustrates the structure of the mixDoC model used to analyze the causal relationships in twin data. The top level represents the entire pool of twins, which is then divided into monozygotic (MZ) and dizygotic (DZ) twin pairs. Each of these groups is further split based on causal concordance or discordance. Positive signs (+) indicate that the causal direction is from X to Y, while negative signs (−) indicate that the causal direction is from Y to X.

The model developed here is a mixture distribution DoC twin model (mixDoC), which includes two classes that differ in the direction of the causal relationship between the two traits, X and Y. The two classes correspond to subpopulations characterized by opposite causality directions. In one subpopulation, X causes Y, while in the other, Y causes X. Since the DoC model uses pairs of twins as the sampling unit and each twin could be drawn from either causal distribution, there are four possible combinations for each family: two combinations where the twins are concordant for causal direction and two combinations where they are discordant. Specifically: 1) X causes Y in both twins; 2) Y causes X in both twins; 3) X causes Y in twin 1 and Y causes X in twin 2; or 4) Y causes X in twin 1 and X causes Y in twin 2. Since the ordering of twins within a twin pair is usually random or unsystematic, the expected parameter estimates and mixing proportions of the discordant classes are expected to be equal. Figure 2 shows a schematic of the model. The model consists of two groups (MZ and DZ twin pairs), with four mixture component classes within each group.

The ability to disaggregate mixtures of opposing causal processes stems, in large part, from differences in the observed means that arise from each causal process. To identify the DoC model within each concordant class, means for each trait (X and Y) are equated across twin 1 and twin 2 and across zygosities. However, the causal process may differ between the classes, thus altering the expected mean for each variable. The means in the class where X causes Y may differ from the means in the class where Y causes X. This distinction results in two means for each trait, where the phenotypic mean differences across classes are associated with the direction of causation. For model identification, at least two of the parameters, $b_{x->y},\;b_{y->x},\;r_{A},\;r_{C}$, or $r_{E}$_,_ must be fixed to a specific value, which is zero in this case. Constraining all three parameters to equal zero specifies that there is no latent confounding due to genetic, shared environmental, or unique environmental sources. In that case, the only source of phenotypic covariance is the causal relationship. To limit the set of data-generating models, we considered two models: one in which all background confounding was set to zero (i.e., $r_{A}=r_{C}=r_{E}=0$) for all classes and unidirectional causation, and an extended model where the mixture still focused on unidirectional causation but also allowed for background genetic confounding (r_A_ was freely estimated but equated across class).

### Simulation Design Parameters

All analyses were performed using R version 4.2.1 (R Core Team, 2021) with models fitted using OpenMx v2.20.6 (Neale et al., 2016). Code is available in a Github repository (https://github.com/Pvinh147/mixDoC). All data were simulated under a bivariate Gaussian mixture model, in which the data are generated from a finite mixture of bivariate Gaussian distributions.

For each simulation, eight separate datasets (four classes for each zygosity group) were generated and merged into MZ and DZ twin datasets. An overview of the simulation designs are provided in Table 1. To model different degrees of heterogeneity, simulations were completed with varying proportions of concordant and discordant twin pairs at fixed parameter values. These values were chosen to explore the feasibility of this mixture model rather than to exhaustively explore the multidimensional space.

A measure of entropy was used to evaluate the accuracy with which we can probabilistically assign individuals to classes based on the results of fitting the mixDoC model. The relative entropy index (Ramaswamy et al., 1993) is expressed as:

2 $$Ent=1-\frac{\sum_{i=1}^{N}\;\;\sum_{i=C}^{C}\;\;-p_{iC}\ln p_{iC}}{\mathrm{Nln}C}$$

where C is the number of classes, $p_{ic}$ is the estimated posterior probability for individual i in class C, N is the number of observations. Entropy values range from zero to one, where values closer to one indicate better classification, meaning that the posterior probabilities approach zero or one. To evaluate the entropy of the mixture model, we used parameter estimates averaged from 1,000 replications. Parameters that we varied include: i) strength of the causal effects; ii) class means; iii) modes of inheritance; and iv) presence of genetic confounding.

We fitted the following models to each dataset: i) the novel 4-class-per-zygosity mixture distribution (4-class); ii) the concordant-pairs-only reduced mixture model (2-class); iii) non-mixture X causes Y DoC; iv) non-mixture Y causes X DoC; v) non-mixture bidirectional DoC; and vi) non-mixture bivariate ACE model with *r*_*A*_, *r*_*C*_, and *r*_*E*_ estimated, i.e., trait covariance is entirely due to the sharing of A, C, and E factors.

## Results

### Model Fit Statistics

Model fit statistics for the mixture and non-mixture direction of causation twin models are presented in Table 2. The novel 4-class-per-zygosity mixDoC model and the competing models mentioned above were fitted to data with varying degrees of heterogeneity in the true data-generating model.

### Equal Proportions Scenario

Table 2A compares the fit statistics across the DoC models under the condition where there are equal numbers of twin pairs exhibiting concordance from X to Y, Y to X, and discordance for direction of causation (25% of each of the four mixture classes). The AIC of 144325.4 for the data-generating model is the lowest, which is expected. The substantial difference in AIC, approximately 4000, indicates that with a sample size of 1,000 pairs, distinguishing between the mixture and non-mixture models yields informative results. This suggests that the mixDoC model can effectively capture the heterogeneity present in the data.

### Additional Scenarios

Supplementary Table 1 reports results for additional scenarios with different proportions of concordant and discordant twin pairs. Overall, when data heterogeneity due to differing causal directions at the subpopulation level is present, the mixDoC models show greater parsimony, reflected by the lower AIC values. Conversely, when data are simulated from a single distribution, such as the X causes Y DoC model (applicable uniformly across all zygosity pairs), the mixDoC model fit statistics demonstrate less parsimony (higher AIC values) compared to other DoC models.

### Bidirectional Causation Scenario

Table 2B compares the fit statistics across the DoC models when the data-generating model is the bidirectional DoC model for all twins, where no mixture distribution is present. In this scenario, the mixDoC models show less parsimony compared to the bidirectional model. This difference is evident in the AIC column of Table 2B, where the AIC of 45953.44 for the bidirectional model is lower (indicating better fit) than 45955.44 for the Cholesky model and much lower than the 4-component mixture model AIC of 45986.20. This suggests that in the absence of mixture distribution, simpler models may provide a better fit.

### Entropy and Classification Accuracy

Table 3 presents entropy values for simulations assessing the impact on classification when varying the class-specific means, causal effect size, trait heritability, or genetic confounding. Entropy is a measure of classification accuracy, with higher values indicating better classification.

### Impact of Phenotypic Means

The results indicate that entropy depends heavily on the phenotypic means. Phenotypic mean differences refer to the average differences in the same trait between different groups. In this study, it means the difference in the average levels of a trait (e.g., depression) between subpopulations with different causal directions (e.g., X causes Y versus Y causes X).

Larger mean differences within the same trait across groups lead to higher entropy, reflecting better classification accuracy. For instance, if individuals in the X causes Y group have significantly higher levels of trait X than those in the Y causes X group, the model can more reliably classify individuals based on their trait levels. As shown in Table 3, increasing phenotypic mean differences results in higher entropy values, indicating improved classification accuracy.

This highlights the importance of phenotypic mean differences in the performance of the mixDoC model. Substantial phenotypic mean differences enable the model to accurately identify underlying causal structures, leading to precise estimates of causal effects and better classification of individuals.

### Impact of Causal Effect Size

The results show that larger causal effect sizes improve classification accuracy. This is because stronger causal relationships create clearer distinctions between groups, making it easier for the model to classify individuals accurately. As indicated in Table 3, higher causal effect sizes correspond to increased entropy values, demonstrating better classification accuracy.

### Impact of Trait Heritability and Genetic Confounding

Classification accuracy deteriorates when the proportions of variance between the two traits become more similar. Additionally, the presence of covariance between the background A, C, or E factors for the two traits negatively impacts entropy, indicating that genetic and environmental confounding can obscure the causal relationships being modeled.

## Discussion

The model developed and tested in this article integrates the Direction of Causation (DoC) twin model with finite mixture modeling to address potential population heterogeneity due to differences in causal directions. Our results demonstrate that in the presence of data heterogeneity, mixture models exhibit better fit statistics, even with low levels of data heterogeneity. Conversely, the mixDoC twin model is less parsimonious when the population is homogeneous or when the causal effect is bidirectional. This finding underscores the importance of considering population heterogeneity in causal modeling, particularly in psychiatric and behavioral genetics where diverse causal pathways are common.

As with all models, the mixDoC has limitations. Similar to the DoC model, it assumes random mating, no genotype-environment interactions, and no genotype-by-environment covariance. These assumptions simplify the model but may not hold true in all real-world scenarios. Both models are susceptible to measurement error, which may bias estimates of the causal effect if the amount of error differs substantially between traits. Measurement error is a critical issue in psychological and medical research, where precise measurement of constructs is challenging.

A prominent limitation of the mixDoC model, common to other finite mixture models, is the need for large sample sizes to obtain accurate classification. This requirement arises because the model’s classification accuracy depends on large class separation, driven by phenotypic mean differences between classes and the magnitude of the causal effects. Given that means have much smaller standard errors than variances and covariances, the covariance structure from a mixture of causal directions differs little from that under homogeneity models, making detection difficult without large sample sizes. This highlights a common trade-off in statistical modeling between model complexity and the power to detect effects.

A second limitation is the assumption that within each mixture class, the data are distributed as multivariate normal. Departures from multivariate normality, such as those induced by scaling artifacts, may lead to incorrect inferences. Several of these assumptions can be tested with additional data. For instance, the assumption of no A-C covariance can be tested by adding polygenic scores for the two traits to the model (Dolan et al., 2021). Random mating can be assessed by including marital pairs assessed on the same traits. Genotype-by-environment interactions may be tested at the observed level by including interacting variables (Purcell, 2002), ideally exogenous causes of the traits to avoid collider bias. GxE interaction at the latent level can be assessed when variables are measured continuously and multiple indicators of latent traits are available. Measurement error can be evaluated with test-retest protocols, ensuring an appropriate inter-test interval to reduce interference but avoiding true developmental change.

Another limitation is the model’s distributional assumption of multivariate normality for class distributions. While such data may be available for neuroimaging measures, biochemical assays, or physical traits, well-behaved data are scarce in the behavioral realm and often derived from questionnaires or direct behavioral observations. Departures from multivariate normality due to imprecise assessments could spuriously generate apparent evidence for a mixture distribution when none exists. Analyzing at the latent trait level could be a potential approach in such cases. Future research could explore alternative distributions or non-parametric approaches to address this issue.

Despite these limitations, the analyses show that the mixDoC model can correctly recover both parameter estimates and mixture proportions. Even with modest heterogeneity, the mixture model demonstrates greater parsimony than the unidirectional and bidirectional DoC models. Aside from requiring sufficient phenotypic mean differences according to which trait is causal, the model is viable. The mixture model not only detects latent heterogeneity but also estimates individual conditional class membership probabilities. This ability to assign probabilities to individuals provides a powerful tool for personalized analysis and treatment planning.

Although this method seems limited to pairs of relatives, it could, in principle, be used to assess the probability that a randomly selected individual or patient belongs to one or other of the mixture classes. Longitudinal data would be valuable in this context, with occasion 1 and occasion 2 serving as proxies for twin 1 and twin 2 to uncover latent population heterogeneity. A mixture distribution model for the cross-lagged panel design would be similar to the twin model presented here but would lack MZ and DZ groups, losing the ability to differentiate between individual-specific and familial sources of variation. However, the cross-lagged panel model could still offer valuable insights into the temporal dynamics of causal relationships.

Determining the causal direction at an individual level through calculating individual posterior class membership probabilities is potentially valuable in deciding on a course of treatment. In a therapeutic setting, patients undergoing the same treatment often experience variability in outcomes. One possible source of limited response to treatment may be differences in causal direction. Treating the condition causally downstream may provide temporary relief but lack lasting effects due to the continued presence of the upstream cause. Conversely, treating the upstream causal variable should improve both disorders. This approach aligns with the precision medicine paradigm, which aims to tailor medical treatment to the individual characteristics of each patient.

In conclusion, by integrating the Direction of Causation twin model with finite mixture distribution, we developed a model that accommodates heterogeneity due to subpopulations differing by causal direction. The mixDoC model has the potential to contribute towards explaining heterogeneity in treatment outcomes. While currently limited to multivariate normally distributed bivariate twin data, future developments could incorporate additional family members, multivariate, and longitudinal data. With multivariate data, specifying that the mixture distribution operates at the level of a latent factor with multiple indicators is possible. This approach could overcome the need for data to conform to the multivariate normal distribution within each mixture component class, making it more applicable to behavioral and psychological measures. Further research should explore these extensions and evaluate their performance in diverse empirical contexts.

## Figures and Tables

**Table 1:** Simulation Designs: Table outlines the various parameters used in the simulation and their corresponding explanations.

Scenario	Parameter	Explanation
Degree of Heterogeneity	Proportion of twins within each class	To determine if the level of heterogeneity affects the classification accuracy.
Presence of Twin Pairs Exhibiting Bidirectional Causation	Proportion of twins exhibiting Bidirectional Causation	To assess the impact of bidirectional causation on model performance.
Phenotypic Mean Difference	Difference in trait means across groups	To evaluate how differences in phenotypic means between groups influence classification accuracy.
Causal Effect Size	Magnitude of causal relationship	To examine the effect of varying causal effect sizes on model accuracy.
Trait Heritability	Proportion of variance due to genetics (*A*)	To understand how heritability affects the model’s ability to classify individuals.
Genetic Confounding	Presence of genetic confounding (*ra*)	To investigate the impact of genetic confounding on model classification accuracy.

**Table 2:** Model fit statistics for Causal Models under Different Data Generating Scenarios: Table presents the model fit statistics for various causal models under different data generating scenarios. The models include a 4-class mixture, 2-class mixture, unidirectional DoC (X → Y and Y → X), bidirectional, and Cholesky models. Fit statistics include degrees of freedom (df), minus twice the log-likelihood (−2LL), and Akaike Information Criterion (AIC).

A. Data Generating Model: Unidirectional (X → Y, Y → X, Discordant), 5000 MZ and 5000 DZ twin pairs each			
Model	df	−2LL	AIC
4-Class mixture	119984	144293.4	**144325.4**
2-Class mixture	119986	148372.6	148400.6
DoC (X → Y)	119991	148864.1	148882.1
DoC (Y → X)	119991	148534.6	148552.6
Bidirectional	119990	148469.6	148489.6
Cholesky	119989	148435.8	148457.8
B. Data Generating Model: Bidirectional, 5000 MZ and 5000 DZ twin pairs			
Model	df	−2LL	AIC
4-Class mixture	39984	45854.20	45986.20
2-Class mixture	39986	45954.16	45982.16
DoC (X → Y)	39991	46635.55	46653.55
DoC (Y → X)	39991	46060.20	46078.20
Bidirectional	39990	45933.44	**45953.44**
Cholesky	39989	45933.44	45955.44

**Table 3:** Entropy Values under Different Simulation Conditions. Data were simulated for 5000 twin pairs with class proportions equal across classes and zygosity. Each section of the table varies only the specified parameter while all other parameters are held constant. For the section, Additive Genetic Variance of Trait Y, the additive genetic variance of X (*a_X_*) is fixed at 0.7.

Phenotypic Mean Difference			Causal Effect Size		
X	Y	Entropy	b_12_	b_21_	Entropy
0.1	0.1	0.209	0.1	0.1	0.210
0.5	0.1	0.384	0.1	0.5	0.283
1.5	0.1	0.738	0.1	0.8	0.337
0.5	0.5	0.701	0.5	0.5	0.432
1.5	1.5	0.998	0.8	0.8	0.594
Additive Genetic Variance of Trait Y			Genetic Confounding		
*a_y_*		Entropy	*ra*		Entropy
0.1		0.286	0.1		0.260
0.3		0.238	0.15		0.249
0.4		0.233	0.20		0.238
0.5		0.231	0.25		0.237
0.6		0.230	0.3		0.230

## Data Availability

R coding scripts for fitting the mixCLPM model can be found on GitHub at https://github.com/Pvinh147/mixDoC.
